# Flea-Borne Typhus Presenting with Acalculous Cholecystitis and Severe Anemia

**DOI:** 10.1155/2023/5510295

**Published:** 2023-11-03

**Authors:** Ramya Varadarajan, Ashmi P. Patel, Keyvon Rashidi, Albert Oh, Rashmeen Rahman, Ryan Neal

**Affiliations:** ^1^Texas A&M College of Medicine, Houston Methodist Hospital, Houston, TX 77030, USA; ^2^Department of Medicine, Houston Methodist Hospital, Houston, TX 77030, USA; ^3^Department of Endocrinology, University of Texas Medical Branch, Galveston, TX 77555, USA

## Abstract

**Background:**

Flea-borne typhus (FBT), an uncommon illness in the United States, typically presents as a high continuous fever with commonly associated symptoms including headache, myalgias, and rashes on the trunk and extremities. Patients infected with FBT may also present with atypical symptoms. As such, the combination of its relatively low incidence in the United States coupled with its variability in associated symptoms poses a diagnostic challenge for clinicians; early empiric treatment with doxycycline is warranted prior to a definitive diagnosis to reduce the risk of damage to vital organs. *Case Report*. This case describes a 54-year-old male who presented to an emergency room in Houston, Texas, with one week of constant right upper quadrant abdominal pain and fevers up to 40°C. The patient was initially diagnosed with Grade III severe acute cholangitis after abdominal ultrasound revealed gallbladder sludge and wall thickening without ductal dilatation, but a subsequent endoscopic retrograde cholangiopancreatography was unremarkable. Following intermittent fevers and worsening anemia, the patient was started on oral doxycycline for atypical infection, and an infectious disease workup subsequently returned a positive titer for *Rickettsia typhi*. He experienced rapid symptomatic and clinical improvement, and the patient was discharged home with a final diagnosis of flea-borne typhus.

**Conclusion:**

Albeit uncommon, the presentation of this patient's symptoms and final diagnosis of flea-borne typhus demonstrates the importance of (1) keeping atypical infections such as FBT in the differential diagnosis and (2) beginning empiric treatment to prevent damage to vital organs if suspicion of FBT is high.

## 1. Introduction

Flea-borne typhus (FBT) typically presents as high, continuous fevers with variable general associated symptoms; the most common presentation includes headache, myalgias, and rashes on the trunk and extremities [1]. Although flea-borne typhus fever is not a common infection in the United States, cases are often concentrated in areas where its arthropod vectors (various flea species, especially the cat flea, *Ctenocephalides felis*) thrive, including Texas, California, and Hawaii. The nonspecific symptoms of typhus fever often lead clinicians to investigate more common causes for infection, such as pulmonary, cardiac, hematologic, and hepatobiliary sources prior to considering atypical causes, especially in patients with no clear risk factors aside from geographic location. Following infection by *Rickettsia typhi* and phagocytosis by dendritic cells, replication occurs in the lymph nodes and the bacteria enter the bloodstream [2]. Following the hematologic spread of the infection, endothelial injury occurs with a subsequent increase in vascular permeability, resulting in symptoms ranging in severity from rashes to acute kidney injury, pneumonia, meningoencephalitis, multiorgan failure, and death [3]. Empiric treatment with doxycycline is warranted when *R*. *typhi* infection is suspected prior to definitive diagnosis with serological testing, as early treatment reduces the risk of damage to major organs, including the lungs, kidneys, liver, brain, and heart [4]. Here, we report a case of FBT presenting with atypical symptoms, including right upper quadrant abdominal pain, direct hyperbilirubinemia, and anemia to highlight the diagnostic challenges surrounding FBT.

## 2. Case Summary

A 54-year-old male with no significant past medical history presented with one week of right upper quadrant abdominal pain and fevers up to 40°C. The abdominal pain was constant, worsened by positional changes and exertion, and unrelated to eating. A review of systems was significant for mild upper respiratory symptoms, anorexia, nausea, and vomiting. On presentation to an outside emergency room in Houston, Texas, the patient was febrile at 39.4°C and tachycardic but otherwise hemodynamically stable. Due to the patient's upper respiratory symptoms and signs of consolidation on chest imaging, the patient was started on azithromycin and ceftriaxone for suspected pulmonary infection. Upon transfer to the hospital, the patient remained febrile and tachycardic, and a physical exam revealed scleral icterus and right upper quadrant tenderness with no rebound tenderness, guarding, or rigidity. He denied any exposure history to host animals of fleas, including cats, rats, dogs, and opossums, any history of recent travel, or recent exposure to sick contacts.

**Figure 1:**
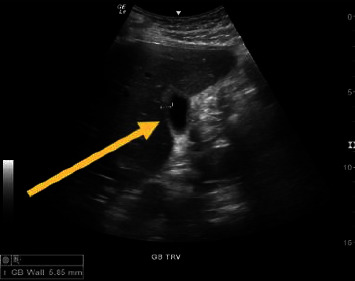
Right upper quadrant abdominal ultrasound on admission showing gallbladder wall thickening to 5.85 mm (indicated by yellow arrow; normal gallbladder wall thickness is less than 3 mm) and no gallbladder stones.

Initial laboratory studies revealed direct hyperbilirubinemia (5.7 mg/dL, reference range (RR) 0–0.3 mg/dL), moderate thrombocytopenia (73 K/uL, RR 150–400 K/uL), elevated liver enzymes (ALT 114 U/L (RR) 5–50 U/L); AST 115 U/L (RR 10–50 U/L); GGT 57 IU/L (RR 0–59 U/L), and elevated lactic acid (2.3 mmol/L, RR 0.5–2.2 mmol/L). Severe normocytic anemia was noted with a hemoglobin level of 10.0 g/dL (RR 14–18 g/dL). The leukocyte count and levels of alkaline phosphatase and amylase were unremarkable. A COVID-19 PCR test was negative. Right upper quadrant abdominal ultrasound revealed minimal gallbladder sludge and wall thickening without ductal dilatation (Figure 1).

The patient was diagnosed with Grade III severe acute cholangitis, started on piperacillin-tazobactam, and evaluated further with repeated hepatic function panels, a hepatitis panel, and magnetic resonance cholangiopancreatography (MRCP). Due to the resolution of the patient's mild upper respiratory symptoms and an identified hepatobiliary source of infection, azithromycin and ceftriaxone were discontinued.

MRCP results were consistent with ultrasound findings, but endoscopic retrograde cholangiopancreatography (ERCP) showed no stones, sludge, pus, or other indications of biliary pathologic changes, and the hepatitis panel was unremarkable. Four days after admission, the patient's abdominal pain began resolving, but he continued to be intermittently febrile and to have worsening anemia with a hemoglobin of 6.2 g/dL, requiring a transfusion of two units of packed red blood cells. He also reported dark stools at this time, raising concern for a post-ERCP gastrointestinal bleed. An esophagogastroduodenoscopy (EGD) performed at the time was negative for any sources of bleeding, raising suspicion that the patient experienced an episode of self-resolved GI bleeding. The patient's hemoglobin level improved to 7.6 g/dL after transfusion and stayed stable at this value until discharge; he did not experience any more dark stools. The possibility of hemolysis was ruled out given stable indirect bilirubin levels.

At the same time as the patient experienced an acute drop in hemoglobin, he exhibited worsening direct hyperbilirubinemia and new leukocytosis that was not present on admission, prompting the addition of cefepime and metronidazole to his antibiotic regimen and further infectious disease evaluation. Blood cultures, initially drawn at the outside emergency room, continued to be negative. At this time, empiric treatment for atypical infection, oral doxycycline 100 mg twice daily with meals, was begun, and infectious disease workup subsequently returned a positive titer for *Rickettsia typhi* IgM at >1 : 512 (RR <1 : 64) and IgG at >1 : 1024 (RR <1 : 64). Therefore, doxycycline was continued and all other antibiotics were discontinued. Within three days after the initiation of doxycycline, the patient showed rapid clinical improvement with resolution of abdominal pain, resolution of fevers, improvement of direct bilirubin levels, and leukocytosis. His hemoglobin level remained stable at 7.9 g/dL upon discharge. Doxycycline 100 mg twice daily with meals was continued after discharge for a total course of 11 days, and the patient was discharged home with a final diagnosis of FBT.

## 3. Discussion

Flea-borne typhus (FBT) is an infection caused by *Rickettsia typhi* after a flea bite, inoculation of a skin abrasion with infected flea feces, or inhalation of aerosolized infected flea feces. Flea-borne typhus is the most prevalent form of typhus in the United States; infections are concentrated in Texas (with an average of 300 cases per year in the 2010s), Hawaii (with an average of 7 cases per year), and California (with >100 probable or confirmed cases per year as per the California Department of Public Health) [4]. This is attributed to the presence of a warmer or more tropical climate, in which exposure to arthropods is more common [5]. Modifiable risk factors include environmental sanitation near the home, flea protection for pets, and reduced exposure to stray and feral dogs and cats [6, 7].

The typical presentation of FBT includes fevers (>95% of patients), headaches (80.9%), myalgias (51.5%), rash (47.5%), and back pain (35%). Gastrointestinal/abdominal symptoms of typhus include nausea and vomiting (26.7%), hepatomegaly (22.1%), splenomegaly (16.8%), diarrhea (18.6%), and abdominal pain (18.1%) [8]. Among our patient's symptoms, localized right upper quadrant abdominal pain and upper respiratory symptoms were not typical for cases of FBT, further attesting to typhus fever's vast variety of potential presentations. In patients in the United States, two older studies had shown a 0.3% occurrence of jaundice in cases of murine typhus [9, 10]. More recent studies indicate a rising incidence of 3–11% of patients presenting with jaundice in cases of FBT [11, 12].

In our case, the source of infection was initially thought to be pulmonary in nature due to the upper respiratory symptoms in conjunction with findings on chest imaging. The persistence of the patient's fever and abdominal pain and the quick resolution of respiratory symptoms, however, favored an alternate diagnosis. In addition, further evaluation revealed direct hyperbilirubinemia with signs of hepatic dysfunction. Given these findings, a hepatobiliary cause was suspected and further supported with findings of Grade III cholangitis on right upper quadrant abdominal ultrasound. However, negative ERCP findings and lack of clinical improvement after the initiation of broad-spectrum antibiotics effectively ruled out a biliary cause of infection. Other atypical causes, including treponemal infection, legionellosis, leptospirosis, and FBT fever were considered at this point; FBT was considered as a potential diagnosis due to its broad range of symptoms and potential abdominal involvement. The patient's improvement on oral doxycycline was supportive of a diagnosis of FBT, which was confirmed by a positive *Rickettsia typhi* titer. The patient's acute drop in hemoglobin during his hospital course was likely linked to a self-resolving case of gastrointestinal bleeding after ERCP, but the presence of normocytic anemia on admission and discharge with no prior noted history of anemia remained unexplained and may have been linked to his diagnosis of FBT.

## 4. Conclusion

In conclusion, the prevalence of FBT has been rising in the United States over the past two decades; however, the specific and unique presentation of symptoms in this case is notable [4]. Acalculous cholecystitis and/or biliary dysfunction in FBT have been reported in three prior case reports and one retrospective chart review. The three case reports also noted the presence of upper respiratory symptoms that were likely linked to FBT given a lack of symptomatic relief with treatment of presumed bacterial respiratory infection [13–16]. Our case further reinforces the presence of upper respiratory involvement in FBT. None of the aforementioned cases noted severe normocytic anemia at the time of diagnosis, as we noted in our case. The clinical manifestations of FBT are often indistinguishable from other acute febrile infectious diseases [11]. If atypical infection is suspected, it may be reasonable to begin empiric treatment for FBT, especially due to the length of time required for the indirect fluorescent antibody serological tests to result [17]. Although the mortality rate of typhus is reported to be only 0.4% globally, prompt empiric treatment with doxycycline can prevent damage to the liver, kidneys, and other organs [18]. As such, the combination of symptoms present in this patient warrants consideration of FBT in the differential diagnosis and empiric treatment with doxycycline for rapid clinical improvement and prevention of organ damage. This patient's FBT infection was effectively treated with a total doxycycline course of 11 days and the patient did not experience any chronic complications.

## Data Availability

Data sharing is not applicable to this article as no datasets were generated or analyzed during the current study.
